# Activation of the Kinin B1 Receptor Attenuates Melanoma Tumor Growth and Metastasis

**DOI:** 10.1371/journal.pone.0064453

**Published:** 2013-05-17

**Authors:** Patricia Dillenburg-Pilla, Andrea G. Maria, Rosana I. Reis, Elaine Medeiros Floriano, Cacilda Dias Pereira, Fernando Luiz De Lucca, Simone Gusmão Ramos, João B. Pesquero, Miriam G. Jasiulionis, Claudio M. Costa-Neto

**Affiliations:** 1 Department of Biochemistry and Immunology, Faculty of Medicine at Ribeirão Preto, University of São Paulo, Ribeirão Preto, Brazil; 2 Departament of Pathology, Faculty of Medicine at Ribeirão Preto, University of São Paulo, Ribeirão Preto, Brazil; 3 Department of Biophysics, Federal University of São Paulo, São Paulo, Brazil; 4 Department of Pharmacology, Federal University of São Paulo, São Paulo, Brazil; The Moffitt Cancer Center & Research Institute, United States of America

## Abstract

Melanoma is a very aggressive tumor that does not respond well to standard therapeutic approaches, such as radio- and chemotherapies. Furthermore, acquiring the ability to metastasize in melanoma and many other tumor types is directly related to incurable disease. The B1 kinin receptor participates in a variety of cancer-related pathophysiological events, such as inflammation and angiogenesis. Therefore, we investigated whether this G protein-coupled receptor plays a role in tumor progression. We used a murine melanoma cell line that expresses the kinin B1 receptor and does not express the kinin B2 receptor to investigate the precise contribution of activation of the B1 receptor in tumor progression and correlated events using various *in vitro* and *in vivo* approaches. Activation of the kinin B1 receptor in the absence of B2 receptor inhibits cell migration *in vitro* and decreases tumor formation *in vivo*. Moreover, tumors formed from cells stimulated with B1-specific agonist showed several features of decreased aggressiveness, such as smaller size and infiltration of inflammatory cells within the tumor area, higher levels of pro-inflammatory cytokines implicated in the host anti-tumor immune response, lower number of cells undergoing mitosis, a poorer vascular network, no signs of invasion of surrounding tissues or metastasis and increased animal survival. Our findings reveal that activation of the kinin B1 receptor has a host protective role during murine melanoma tumor progression, suggesting that the B1 receptor could be a new anti-tumor GPCR and provide new opportunities for therapeutic targeting.

## Introduction

Melanoma is a very aggressive and metastatic tumor that does not respond well to standard therapeutic approaches, such as radio- and chemotherapies. Consequently, patients diagnosed at late stages of the disease often have a very poor prognosis, with survival averaging approximately 8 months [1]. In fact, the acquisition of a metastatic phenotype is directly related to incurable disease in many types of tumors, making tumor metastasis the main cause of death in cancer patients. To reach secondary organs, tumor cells must acquire the ability to detach from the primary site, invade the host stroma and reach lymphatic or blood vessels. Once in the circulation, tumor cells must still evade the host immune response, survive in the absence of cell attachment, and be able to adhere and trans-migrate through the endothelium to reach the target organs [2]. Although the mechanisms behind each of these multi-step processes are not completely elucidated, it is known that many tumor cells can hijack the physiological function of G-protein-coupled receptors (GPCR) and take advantage of their multiple functions to proliferate, promote angiogensis, evade immune response and invade host tissues to colonize secondary organs [3]. However, emerging information suggests that host cells deploy counterbalance mechanisms to avoid tumor dissemination. To date, a small number of metastatic suppressor genes have been described that significantly reduce metastasis *in vitro* and *in vivo* [4]–[5]. One of these metastasis suppressor genes is KiSS1, the precursor of the ligand for the Gαq – protein coupled receptor GPR54 [6], which was originally shown to inhibit melanoma metastasis [4] and more recently to inhibit CXCR4-mediated chemotactic response in breast cancer cells [7] and endometrial cancer metastasis [8].

Kinins are important inflammatory mediators that are involved in many pathophysiological processes. The kallikrein-kinin system (KKS) response is initiated via the kallikrein-mediated cleavage of kininogen into bradykinin (BK) or kallidin (KD). These two peptides are biologically active; however, they can also act as substrates of carboxypeptidases, originating desArg^9^-BK and desArg^10^-KD. The kinin mediated response occurs through the GPCRs known as the B1 and B2 receptors [9], [10]. While the B2 receptor is ubiquitously expressed and has a high affinity for BK and KD, the expression of the B1 receptor is inducible by activation of CREB, AP1 and NFκB transcriptional factors [11], and once expressed, this receptor binds preferentially to des-Arg^9^-BK and des-Arg^10^-KD. While the role of kinin receptors in tumorigenesis is still poorly understood, it has been shown that angiotensin-I converting enzyme (ACE) inhibitors – a widely used family of anti-hypertensive drugs – have anti-tumor properties [12]–[13]. This finding has been mainly attributed to decreased angiotensin-II (AngII) formation, which is an octapeptide that binds to two different G-coupled receptors known as AT_1_ and AT_2_. AT_1_ is the receptor responsible for the classical effects of AngII such as vasoconstriction [14] and has also been shown to play a role in proliferation, tumorigenesis and metastasis [15]–[16]. Therefore, most of the anti-tumor properties of ACE inhibitors have been eventually attributed to a decreased AT_1_ receptor activation [17]–[18]. However, ACE also has kininase II activity, which means that ACE inhibition not only reduces AngII formation, but also impairs kinin degradation, which ultimately leads to an increase in kinin availability [19]–[20]. The presence of kinin receptors has been reported in several tumors, and a pro-tumor role has been attributed to kinin B2 receptor [21]–[22]. Much less is known about the B1 receptor, although it has been reported that the B1 receptor is up-regulated in pre-malignant and malignant prostate lesions [23] and that its expression is associated with better prognosis in estrogen-negative breast cancer patients [24]. Conversely, it has also been reported that B1 receptor activation induces expression of COX2 [25], MMP-2 and MMP-9 [26] and that B1 receptor specific antagonists diminish proliferation in breast cancer cells [27] and decrease primary tumor growth in lung and prostate xenografts [28] and Ehrlich tumor models [29]. However, a common feature among these previous studies is the expression of the kinin B2 receptor. It has been reported that the B1 receptor may play a pro-tumoral role via cross-talk with the B2 receptor [30].

Considering that ACE inhibition eventually increases kinin availability, that activation of B1 receptor has been reported to inhibit cell migration in normal cells [31], and the fact that the B1 receptor is also a Gαq protein coupled receptor, similar to the metastatic suppressor receptor GPR54, we hypothesized that B1 receptor activation, in the absence of B2 receptor activation, could play a host protective role in tumor progression. In this study, we show that B1 receptor activation inhibits cell migration *in vitro* and decreases tumor growth *in vivo*. Moreover, tumors generated by neoplastic cells that have been stimulated with B1 receptor agonist prior to injection in mice are significantly less aggressive, with a smaller number of tumor cells undergoing cell division, a reduced vascular network and complete absence of metastatic lymph nodes, which ultimately leads to an increase in animal survival.

## Materials and Methods

### Ethics statement

All the experiments were approved and performed in strict accordance with the guidelines of the Ethics Committee on Animal Experimentation from Faculty of Medicine at Ribeirão Preto, University of São Paulo, (CETEA; protocol 025-2007). Mice were bred and housed in a specific pathogen-free facility, with room temperature controlled at 23°C, in a 12 h light/dark circle, and received food and water *ad libitum*. Euthanasia was conducted by cervical dislocation at study endpoint or earlier if animals met any early removal criteria (lethargy, hunched posture, or ruffled coat).

### Materials

The B1 receptor specific agonist DABK was purchased from Sigma. The peptide antagonist desArg^9^ [Leu^8^]-BK (DLBK) was synthesized using the Fmoc solid phase strategy [32], hydrolyzed [33], analyzed to confirm its amino acid sequence by ionic exchange chromatography and purified by HPLC. To confirm its biological activity, we tested the antagonist in a rabbit aorta contraction assay as described previously [34]. All peptides were solubilized in sterile water. DNAse, all PCR reagents and the fluorescent probe for intracellular calcium FLUO-3/AM were purchased from Sigma. Improm II for reverse transcription was purchased from Promega. Anti-phospho-ERK, anti-ERK1/2, horseradish peroxidase-conjugated secondary antibodies and an ECL kit were all purchased from Santa Cruz Biotechnology. B1 receptor antibody was purchased from Abgent and cell culture media and supplements were purchased from Gibco. cDNA coding for B2 receptor, which was used to transfect Tm5 cells was provided by Dr. Joao B. Pesquero [35]. Immunohistochemistry reagents, including anti-Ki67, anti-CD31, secondary antibodies, ABC kit and substrate (3,3′-diaminobenzidine) were purchased from Dako, BD Bioscience, Vector Lab and Sigma, respectively.

### Cell culture

The Tm5 melanoma cell line and the non-tumorigenic melan-a cell line were previously characterized and reported by Dr. Miriam G. Jasiulionis and were maintained in RPMI 1640 pH 6.9 containing 5% FBS and 10 μg/mL of gentamicin, as previously described [36]. The melan-a cells received 200 nM of phorbol 12-myristate 13-acetate (PMA). All experiments using cells were performed using subconfluent cultures (80–90%), and stimulation was performed in serum-free media. Cells were treated with antagonist (DLBK 10 µM) 30 min prior to treatment with the agonist (DABK 1 µM) unless otherwise noted.

### In vivo studies

Tm5 cells were stimulated with vehicle or DABK 1 μM *in vitro* in serum free media for 24 h and then injected subcutaneously in the dorsal superior region of C57/BL6 male mice weighting approximately 25 g. All used drugs, peptides, and medium were removed by extensively washing cells with PBS prior to injection. Each animal received 3×10^5^ Tm5 cells in 100 μl of serum free media. Tumor size and weight were monitored daily.

### Gene expression analysis

Gene expression was analyzed by either semi-quantitative (sqPCR) or quantitative PCR (qPCR). Tumor samples from *in vivo* experiments were immediately frozen in liquid nitrogen and then pulverized before extracting total RNA using Trizol reagent (Invitrogen). One microgram of total RNA was used for DNAse treatment and subsequent reverse transcription using the Improm II protocol. For sqPCR analysis, target genes were amplified using 50 ng of cDNA and Taq platinum DNA polymerase. The amplification procedure consisted of 26 cycles (cyclophilin B) or 40 cycles (all target genes) (1 min−94°C, 1 min−55°C and 1 min−72°C). Samples were loaded into a 1.5% agarose gel stained with ethidium bromide (1 mg/mL). For qPCR, 10–50 ng of cDNA, platinum SYBR green qPCR supermix UDG with Rox and the ABI Prism 7000 sequence detection system were used. We quantified transcripts relative to the housekeeping gene cyclophilin B as described previously [37]. All oligonucleotide primers used in sq and qPCR analyses are listed in table S1 (GAPDH primers were used as described in [38]).

### Western Blotting

Melanoma cells were serum starved for 24 h and received either vehicle or 1 µM of the B1 receptor agonist DABK for 0, 10, 30, 60 or 180 minutes for ERK activation assay, or 24 h to address kinin B1 receptor levels. The cells were later lysed in a lysis buffer consisting of Tris-HCl 10 mM, pH 7.5; NaCl 150 mM; EDTA 1 mM; EGTA 1 mM; SDS 0.1%; Nonidet P-40 1%; 1 mM PMSF, 10 µg/mL leupeptin, 100 µg/mL aprotinin, 10 mM benzamidine, 1 mM NaF, 1 mM sodium orthovanadate, and 1 mM DTT. The lysate was swirled for 30 minutes at 5°C and centrifuged at 12000× g for 15 minutes. The supernatant was subsequently analyzed for protein content. Samples were loaded into 12% acrylamide gels and separated by SDS-PAGE. Next, the proteins were transferred onto a nitrocellulose membrane. The membranes were blocked with BSA 0.1% and incubated with either anti-pERK, anti-ERK or anti-B1 receptor antibodies followed by anti-mouse (pERK) or anti-rabbit (ERK and B1 receptor) secondary horseradish peroxidase-conjugated antibodies. Immunoblots were visualized using an ECL kit and quantified by densitometry using the software ImageJ (http://rsb.info.nhi.gov/ij/).

### Calcium mobilization assay

Fifty percent confluent cells were loaded with the fluorescent probe FLUO3/AM (1 µM for 30 minutes at 37°C) and then kept in a buffer solution containing NaCl 135 mM, KCl 5 mM, HEPES 10 mM, MgCl_2_ 1 mM, glucose 2 mM, and CaCl_2_ 2 mM at pH 7.2. Cells were stimulated with either DABK (1 µM), DLBK (10 µM) or both at the moment of image. Fluorescence imaging experiments were performed with a scanning laser confocal microscope (Leica SP5, Leica, Bensheim, Germany) with a 63X water immersion objective. The fluo-3 fluorescence dye was excited at 488 nm using an argon ion laser, and the emitted fluorescence was measured at 510 nm. Time-course software was used to capture images of the cells (zyt) in the Live Data Mode acquisition. All experiments were done at room temperature (23–25°C).

### Wound healing assay

The protocol described previously was used with minor modifications [39]. Briefly, confluent Tm5 or B16F10 cell cultures were serum starved for 24 h. In experiments that required expression of B2 receptor, cells were transfected 24 h prior to serum starvation using lipofectamine and 1ug of DNA (empty vector or vector cloned with the cDNA coding for the B2 receptor). Monolayers were wounded in a cross shape with a sterile 10 µl pipette tip, washed twice with PBS to remove detached cells and stimulated with either vehicle or DABK (1 µM) in serum free media. The crosses were photographed by phase-contrast microscopy immediately after wounding and after 24 h of healing. All pictures were quantified at least three different points using Image J software to determine the size of the wound. Values from time zero were subtracted from the 24 h measurements to obtain the percentage of closure. In addition, it has been observed that cells lacking E-cadherin frequently break free from the advancing “wall” of cells and migrate into the wound area as lone cells [40], a process known as single-cell migration. These cells were quantified by subtracting the number of lone cells in the wound area before and after 24 h of healing.

### Cell viability assay

MTT (3-(4,5-dimethylthiazol-2-yl)-2,5-diphenyltetrazolium salt) was added to a final concentration of 0.5 mg/mL into the culture media and incubated for 3 h at 37°C. Viable cells are able to reduce MTT, which creates a violet color. The addition of acid isopropanol (0.04 M HCl) solubilizes the reduced product, which can be quantified by measuring the absorbance at 570 nm.

### Histopathological analyses

Tumor samples were collected with 1 cm of adjacent tissue to preserve the tumor micro-environment and fixed in 10% formalin. Paraffin blocks were prepared, sectioned (4 µm) and stained with hematoxylin and eosin (H&E). Slides were analyzed using a Leitz Model Aristoplan microscope (Germany) coupled to a Leica Model DFC280 color camera (Heerbrugg, Germany). Mitotic cells, and vessels from the tumor as well as peritumor macrophages, neutrophils and lymphocytes were quantified at a magnification of 400× across 10 random, non-coincident microscopy fields.

### Immunohistochemistry analyses

Sections from paraffin blocks (4 μm) were dewaxed, endogenous peroxidase was blocked using 3% H_2_O_2_ in ethanol 70% and antigens were retrieved using 10 mM of citric acid. Next, slides were blocked in PBS/BSA 2.5% and first antibody against Ki67 or CD31 were diluted in blocking solution 1∶50 and incubated overnight at 4°C. After washes, slides were incubated with biotinylated anti-rat (Ki67) or anti-mouse (CD31) secondary antibodies 30 minutes at room temperature and ABC kit for another 30 minutes also at room temperature. Color development was performed using 3,3′-diaminobenzidine under microscopic supervision. Once developed, reaction was topped by distillated water washes, and slides were counterstained with Hematoxylin, dehydrated and mounted in permanent mounting media. All slides were scanned using Aperio CS at 400× magnification and quantification was assessed using Aperio algorithms.

### Statistical analyses

Statistical significance was evaluated by either Student's t-test when only two groups were compared or by one-way analysis of variance (ANOVA) using the Student-Newman-Keuls post-test for multiple comparison. Differences between mean values were considered significant when p<0.05.

## Results

### The kinin B1 receptor is functionally expressed in Tm5 melanoma cells

**Figure 1:**
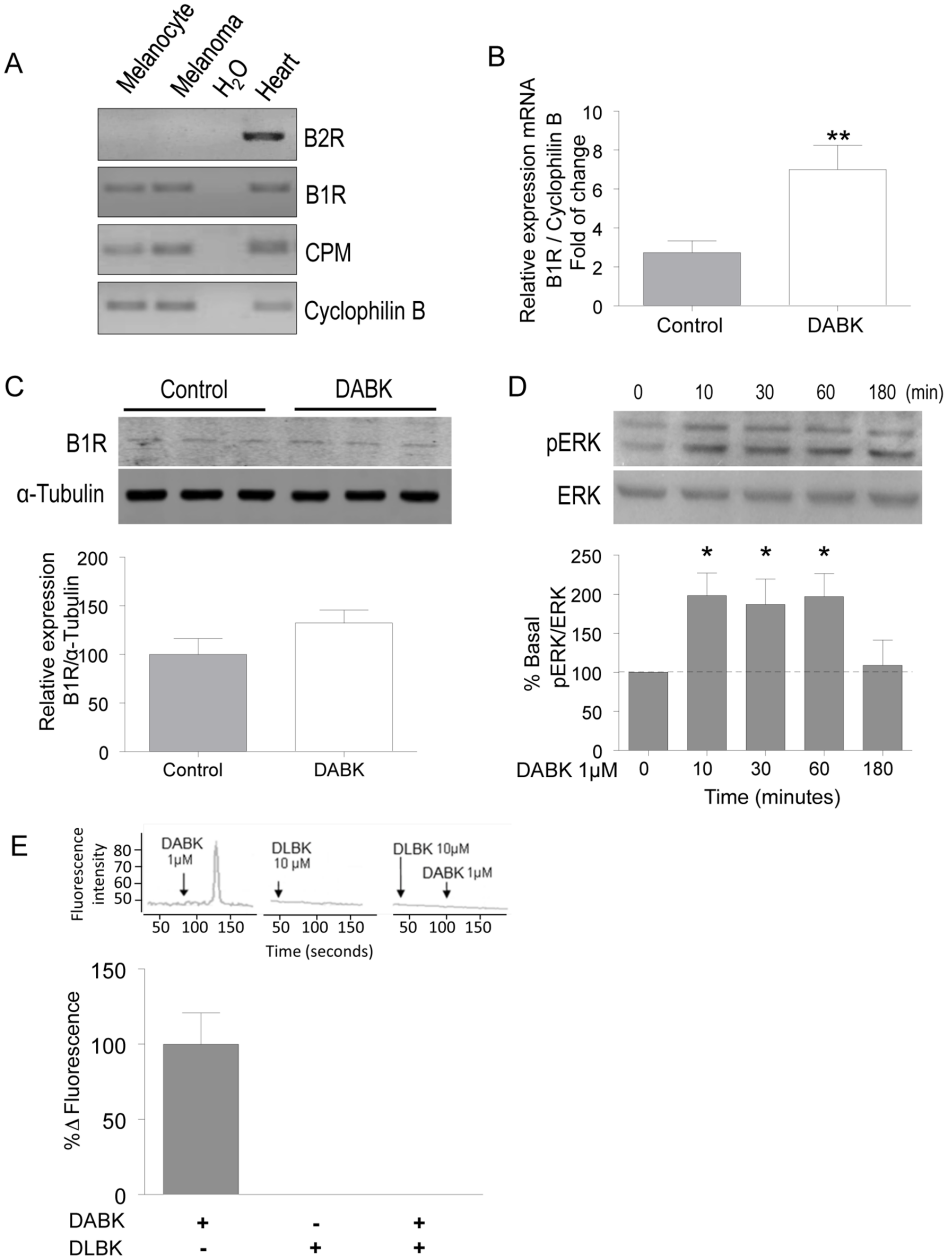
The kinin B1 receptor is functionally expressed in Tm5 melanoma cells. (A) Expression levels of the kinin B1 and B2 receptors, carboxypeptidase M (CPM) and cyclophilin B as a housekeeping gene in melanocytes (melan-a) and melanoma cells (Tm5) evaluated by RT-sqPCR. cDNA from mice hearts was used as a positive control, and water was used as a negative control. (B–C) Kinin B1 receptor is present at protein level in Tm5 cells. Agonist stimulation of the cells has no effect in B1 receptor protein level, however a significant increase in mRNA level was detected. The activity of the B1 receptor in Tm5 cells was accessed by western blotting for ERK phosphorylation (D) and confocal microscopy using FLUO3-AM to evaluate intracellular calcium mobilization (E) after stimulation with the agonist DABK and blockage by the antagonist DLBK. All results are representative or quantification of 3–5 independent experiments. Data are expressed as the mean ± SEM; * p<0.05, with respect to non-stimulated cells; DABK: desArg^9^-bradykinin; DLBK: desArg^9^-[Leu^8^]-bradykinin.

To investigate the contribution of the kinin B1 receptor in tumor progression, we first evaluated the expression of key components of kallikrein-kinin in normal (melan-a) and tumor (Tm5) cells. We used RT-sqPCR to assess the expression of the kinin B1 and B2 receptors. Figure 1A shows that none of the cell lines express the kinin B2 receptor, while they do express the kinin B1 receptor. Both melan-a and Tm5 cell lines also express carboxypeptidase M, one of the main enzymes responsible for the generation of DABK, the selective B1 receptor agonist. In addition, we also confirmed the expression of B1 receptor in Tm5 cells at the protein level by western blotting either at basal state or after agonist stimulation. As seen in figure 1B–C, kinin B1 receptor is present in Tm5 cells and, although its mRNA is up-regulated after DABK treatment for 24 h, we observed no changes in the protein levels after the same time of stimulation. Next, to evaluate if the B1 receptor was functionally active in the Tm5 cells, we evaluated the intracellular pathways that are activated following B1 receptor stimulation with its agonist. We first examined ERK phosphorylation kinetics, obtaining a typical activation profile following agonist stimulation (figure 1D). In addition, we measured intracellular calcium concentration, which rapidly and transiently increased upon agonist stimulation, as expected for a Gαq-coupled receptor, which is completely impaired in the presence of the B1 receptor antagonist (figure 1E). These results indicated that the Tm5 tumor cell line provides the possibility to study the role of the B1 receptor in tumor progression without the interference of B2 receptor signaling.

### Activation of the kinin B1 receptor in the absence of B2 receptor impairs melanoma cells migration in vitro

**Figure 2:**
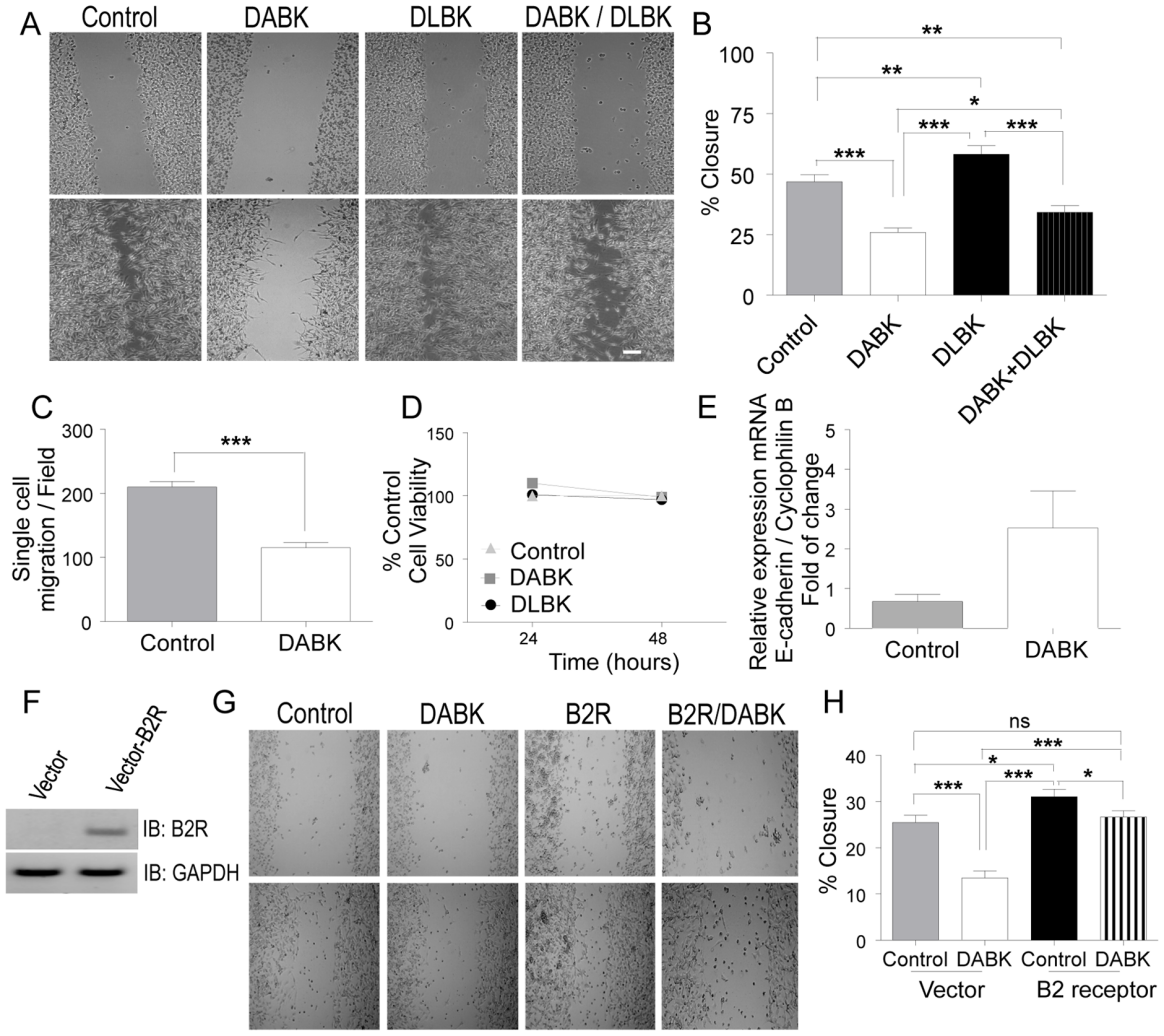
Activation of the kinin B1 receptor in the absence of kinin B2 receptor inhibits melanoma cell migration *in vitro*. Wound healing assays were performed to evaluate the role of the B1 receptor in melanoma cell migration, (A) representative image and quantification of healing (B) and single cell migration (C) of 4 independent experiments in triplicate. (D) Cell viability was accessed using the MTT assay 24 and 48 h after DABK or DLBK stimulation; (E) E-cadherin mRNA expression levels were evaluated by RT-qPCR 24 h after B1 receptor agonist stimulation. Introduction of kinin B2 receptor in B2 receptor-free cells abrogates DABK-mediated migration inhibition (F–H). (All results are from 2–3 independent experiments performed in triplicate, unless otherwise stated). DABK: desArg^9^-bradykinin; DLBK: desArg^9^-[Leu^8^]-bradykinin. Data are expressed as the mean ± SEM; * p<0.05; ** p<0.01; *** p<0.001; **** p<0.0001. The scale bars represent 200 μm.

To address the functional role of the B1 receptor in cellular events related to tumor progression, we first evaluated the effect of its agonist DABK in modulating cell migration. According to the *in vitro* wound healing assay shown in figure 2A–B, incubation with DABK for 24 h leads to ∼50% reduction in wound closure with partial recovery when pre-incubated with the antagonist. Evaluation of the single cell migration profile from the same assay also yielded ∼50% reduction in the group treated with DABK (figure 2C). To rule out the possibility that the observed impairment in migration could be due to a possible cytostatic/cytotoxic effect, we evaluated cell viability up to 48 h in the presence of the aforementioned ligands. As shown in figure 2D, neither incubation with DABK nor with DLBK changed the number of viable cells. In addition, we also observed that the inhibitory effects of B1 receptor activation in cell migration is likely to be related to E-cadherin because activation of the B1 receptor resulted in increased expression of this adhesion molecule (p = 0.0642) (figure 2E). To evaluate a possible cell line specific effect of DABK over Tm5 cell migration, we also evaluated B16F10 cells. Accordingly, B16F10 murine melanoma cells were screened for kinin receptors profile and, as shown in figure S1A, these cells do express B1 receptor and do not have detectable levels of B2 receptor mRNA. Moreover, similarly to Tm5 cells, B16F10 cells showed impaired migration in the presence of DABK (figure S1B–C) as well as reduced single cell migration in the wound healing assay (figure S1D). Interestingly, such inhibitory effect of B1 receptor on cell migration is dependent on the absence of B2 receptor, as expression of B2 receptor in Tm5 cells completely rescued their full migration profile after stimulation with DABK (figure 2F–H).

### Activation of the B1 receptor in pre-implanted tumor cells decreases tumor formation and peritumor inflammatory infiltrate in vivo

**Figure 3:**
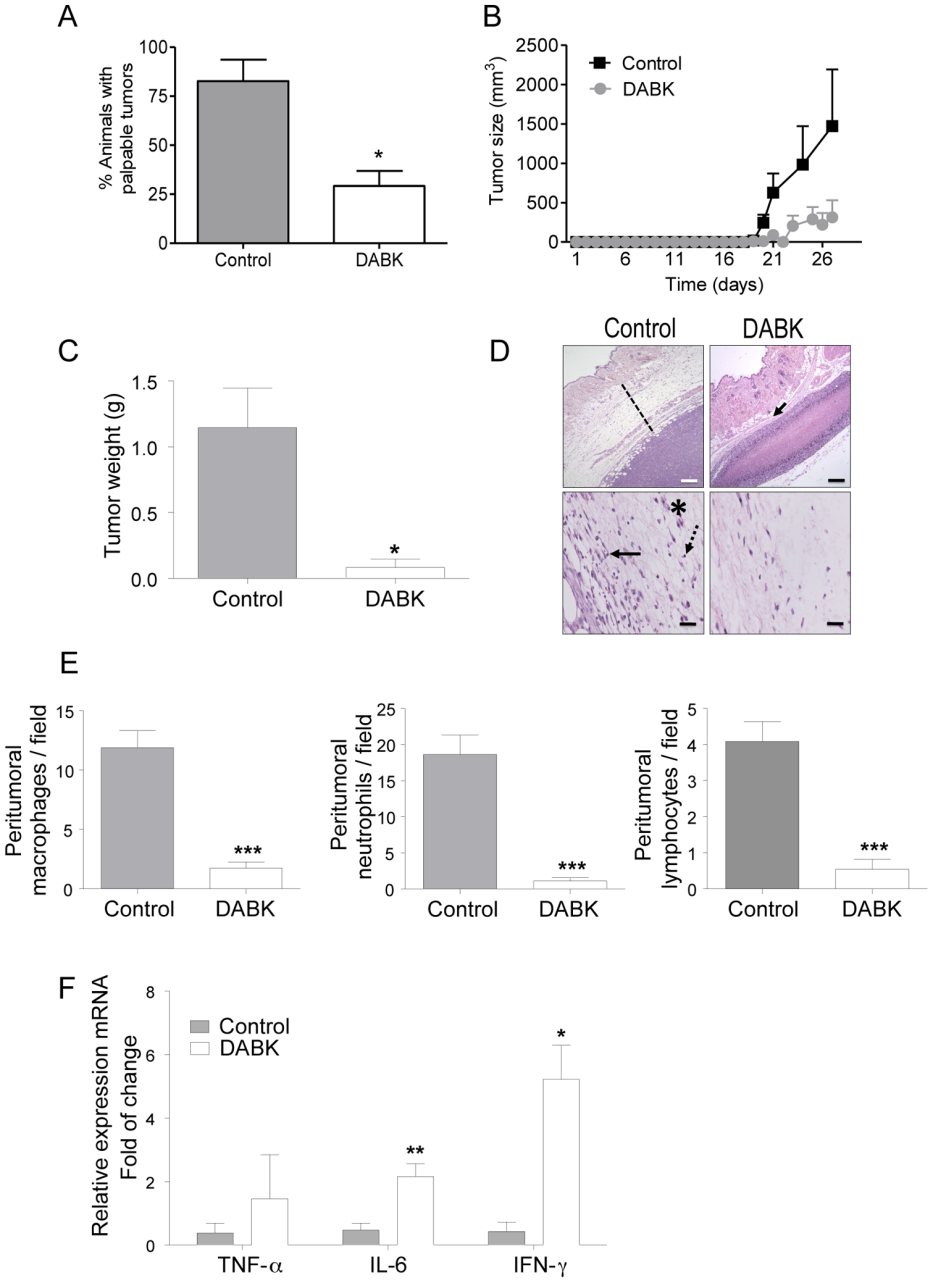
Stimulation of melanoma cells with the B1 receptor agonist reduces tumor growth and peritumor inflammatory infiltration after *in vivo* implantation. (A) Incidence of animals with palpable tumor 28 days after injection of DABK stimulated cells. (B) Tumor growth curve from control and DABK-stimulated cells injected in C57/Bl6 mice. (C) Average tumor weight 28 days after tumor cell injection (D) Representative images at low magnification showing the size of the peritumor inflammatory infiltrate (upper panel, dashed line and arrow) and at high magnification showing the immune cells present in the tumor stroma (lower panel, * macrophage, arrow – neutrophil, dashed arrow – lymphocyte). (E) Peritumor inflammatory infiltrate assessed by quantification of the number of macrophages, neutrophils and lymphocytes in ten different high magnification fields (A = 400x; n = 6). (F) Detection of TNF-α, IL-6 and IFN-γ cytokine expression in the tumor mass as assessed by RT-qPCR (n = 6). *In vivo* studies of primary tumor growth n = 12; Data are expressed as the mean ± SEM; * p<0.05; ** p<0.01; *** p<0.001; DABK: desArg^9^-bradykinin; DLBK: desArg^9^-[Leu^8^]-bradykinin. Scale bars represent 200 μm and 50 μm in the upper and lower panels, respectively.

To evaluate the role of the B1 receptor in tumor progression *in vivo*, we stimulated Tm5 melanoma cells *in vitro* with DABK, the B1 receptor agonist, prior to implantation in mice [16]. While approximately 80% of the animals that received non-treated control cells developed tumors up to 28 days after implantation, only approximately 40% of animals that received DABK-treated melanoma cells developed tumors (figure 3A). In other words, more than 60% of the animals from the DABK-treated group remained tumor-free during this period. Indeed, we monitored tumor size daily from non-stimulated and DABK-stimulated Tm5 melanoma cells engrafted in mice and as seen in figure 3B the growth curves of the control and DABK-tumors are quite different. Although tumors in both groups appear at a similar time-point, DABK-tumors are markedly smaller than control tumors. Likewise, at day 28 after cell implantation, the average tumor mass in the control group was 1.2 g, while in DABK-treated group, the average tumor mass was 0.2 g (figure 3C). The histopathological analysis of the tumors from both groups revealed significant changes in the stroma of the control group but minor changes in the DABK-stimulated tumors (figure 3D, dashed line). Because previous studies have shown that a high number of infiltrated inflammatory cells correlates with a poor prognosis in melanoma [41], we decided to assess whether tumors from DABK-stimulated cells had a lower number of infiltrated immune cells. As shown in figure 3E, there are significantly fewer macrophages, neutrophils and lymphocytes in the area surrounding the tumor in the DABK-stimulated tumors. Nonetheless, even with fewer immune cells, the tumor that originated from the DABK-stimulated cells had higher levels of IL-6 and IFN-γ mRNA (figure 3F), which are pro-inflammatory cytokines known to be important in the anti-tumor immune response [42].

### Melanomas generated from DABK-treated cells show decreased proliferation and vascularization in vivo

**Figure 4:**
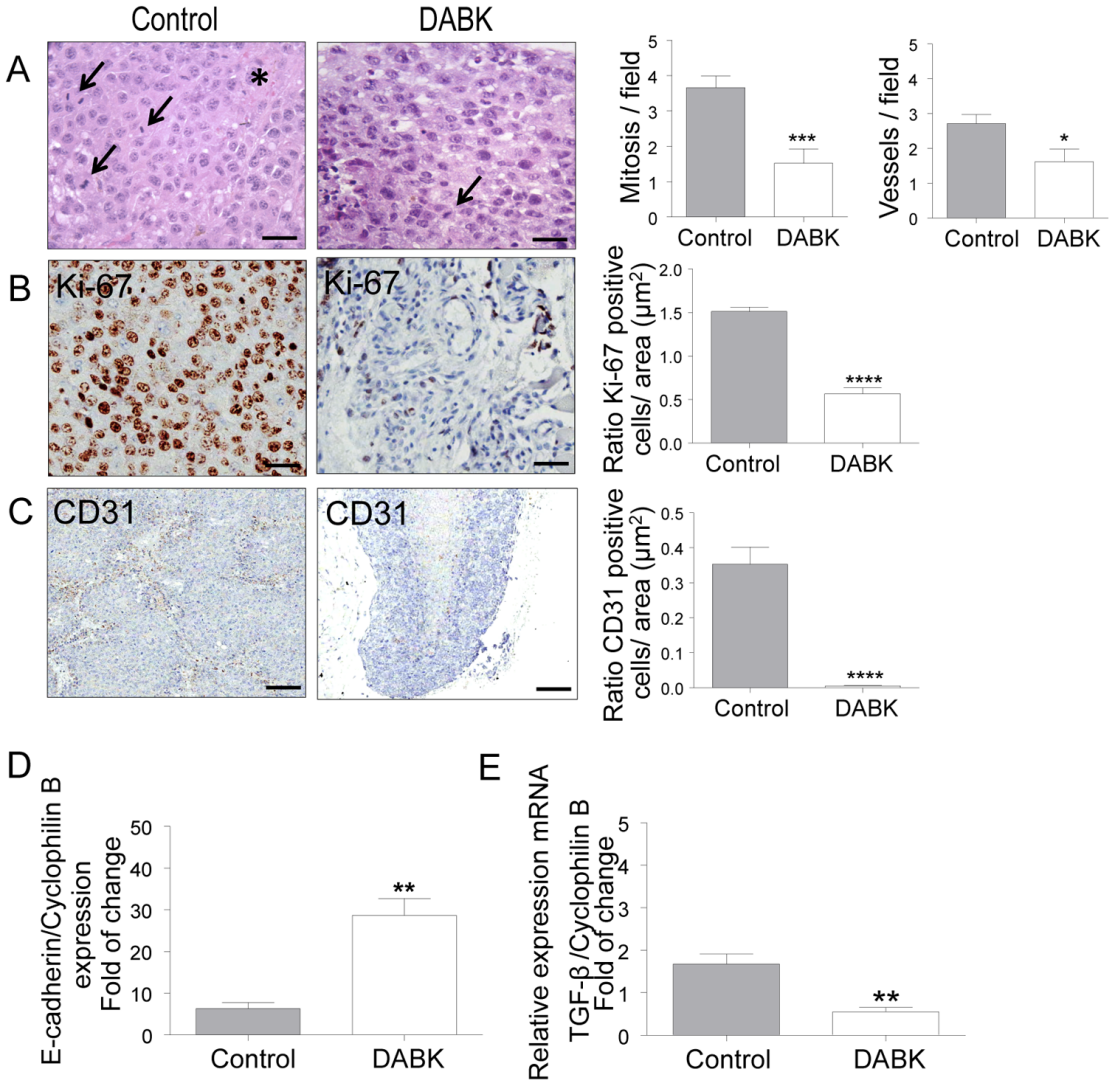
Stimulation of melanoma cells with the B1 receptor agonist decreases tumor proliferation and vascularization after *in vivo* implantation. (A) Histological analysis of tumors generated from non-stimulated or DABK-stimulated melanoma cells (→mitotic cells, ∗blood vessels). Number of mitotic cells and blood vessels were evaluated from ten different high magnification fields (A = 10×40, n = 6). Scale bars represent 50 μm. (B) Immunohistochemistry analysis of proliferation marker Ki-67. Scale bars represent 50 μm (C) Immunohistochemistry analysis of blood vessel marker CD-31. Scale bars represent 200 μm. E-cadherin (D) and TGF-β (E) mRNA expression within the tumor mass was evaluated by RT-qPCR (n = 6). Data are expressed as the mean ± SEM; * p<0.05; ** p<0.01; *** p<0.001; **** p<0.0001; DABK: desArg^9^-bradykinin.

We next performed comparative histopathological analyses with tumor samples from the two groups (control and DABK-treated cells). Tumors generated from DABK-treated cells had significantly less mitotic cells and a poorer vascular network compared to tumors from the control group (figure 4A). Histological findings were further confirmed by immunostaining for proliferation and endothelial markers. In figure 4B, we show representative images and quantification of Ki67 staining in control and tumors generated from DABK-treated cells. The DABK-treated tumors show significant lower levels of Ki67 positive cells as compared to tumors from the control group. A similar scenario is seen in figure 4C, where we show that CD31 staining is also decreased in tumors originated from DABK-treated cells as compared to non-stimulated cells. The ability of tumor cells to grow and metastasize is related to their capacity to undergo morphological/functional changes that will enable those cells to become more motile. In this sense, we assessed expression levels of the adhesion molecule E-cadherin within the tumor mass as well as of TGF-β, a cytokine known for regulating key steps on motility of epithelial cells by inducing EMT. As shown in figure 4D–E, tumors originated from DABK-stimulated cells have higher E-cadherin mRNA levels and lower TGF-β mRNA levels, which correlates with impaired migration capability observed *in vitro* (see figure 2). These results suggest that in addition to impairing primary tumor growth, B1 receptor activation could also play a role in inhibiting metastasis.

### Activation of the B1 receptor in melanoma cells decreases metastasis and increases animal survival

**Figure 5:**
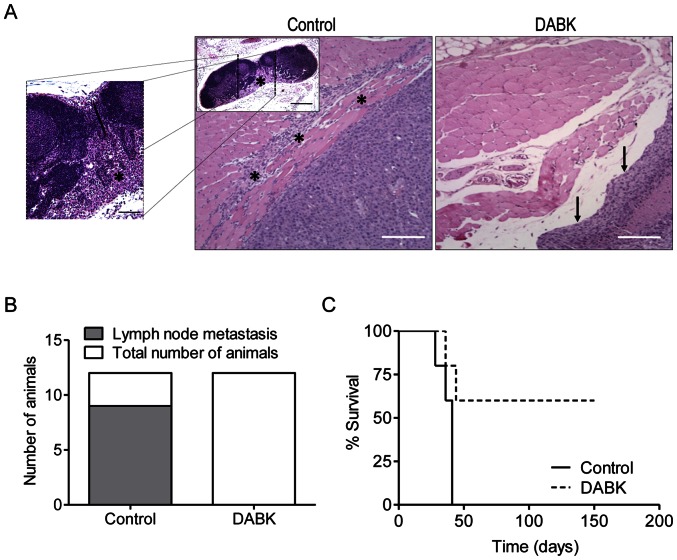
Activation of the B1 receptor in melanoma cells decreases metastasis and increases animal survival. (A) Representative images of the tumor borders showing the invasive behavior of tumors derived from non-stimulated cells and the well-delimited borders of tumors derived from DABK-stimulated cells. The inner panel in the control picture shows a representative image of a metastatic lymph node (* tumor cells invading muscle tissue; → well delimited and encapsulated tumor border). (B) Number of animals with lymph node metastases (C) Survival curve of C57/Bl6 mice that received control or DABK-stimulated tumor cells. n = 12 for lymph nodes metastasis analysis and n = 5 for survival study; Data are expressed as the mean ± SEM; * p<0.05; DABK: desArg^9^-bradykinin.

To address whether B1 receptor activation plays an inhibitory role in tumor progression and metastasis, we performed histopathological analyses that revealed extremely aggressive features in tumors generated from Tm5 melanoma cells. These cells do not show a well-established capsule and frequently invade surrounding tissues, as shown in figure 5A. Moreover, we also observed spontaneous lymph node metastases in 75% (9/12) of the control mice (figure 5B, inner panel and 5A). Conversely, tumors from DABK-treated cells presented a clear capsule delimitating tumor borders, and we did not observe any invasion into the surrounding tissues, as shown in figure 5A, or any lymph node metastases (0/12) in animals implanted with DABK-treated tumor cells (figure 5B).

To evaluate the possibility of late and recurrent onset of disease, we monitored survival up to 150 days after control or DABK-stimulated cell implantation. Strikingly, we observed that animals that did not developed a tumor within the first 30 days after implantation with DABK-stimulated cells were still alive and tumor-free after 5 months, while 100% of animals that received control cells had to be euthanized as late as the beginning of the second month (figure 5C).

## Discussion

The kallikrein-kinin system has a pivotal role in inflammation and vascular permeability systemically. However, many new pathophysiological functions have been attributed to this system since it has been discovered that most of the components of the system can have a local role in different organs, including brain [43]–[45]. The kallikrein-kinin system role in cancer is far from being completely understood, even though the prostate-specific antigen (PSA), which is currently a key marker for prostate cancer diagnostics [46], is in fact a kallikrein. Indeed, several other kallikreins have been described as potential biomarkers for tumor progression [47]–[48]. While recent studies started to uncover the underlying mechanisms by which the kallikrein-kinin system modulates tumor progression, mainly concerning to the B2 receptor, many aspects remain to be elucidated. In this study, we report that the kinin B1 receptor in the presence of its agonist (DABK) and in the absence of the B2 receptor plays a host protective role during murine tumor progression.

The presence of the kinin B2 and B1 receptors in neoplastic tissues has been reported in various tumor cell lines and in patient biopsies [21], [23], [49]. Indeed, it has been shown that blocking the B2 receptor with specific antagonists decreases tumor size, angiogenesis and metastasis [22], [50]–[51] and that the B1 receptor also seems to play a pro-tumor role in prostate cancer [23]. Most of the studies relating the kallikrein-kinin system to cancer were performed in models expressing both the B1 and B2 receptors [23], [25], [29]. It is important to mention that a critical role for the cross talk between the B1 and B2 receptors in kinin-mediated proliferation in androgen-insensitive prostate tumor cells has been reported [30]. Thus, we believe that the reported pro-tumor role for the B1-receptor is most likely due to the cross talking effect with the B2 receptor. Here, we demonstrate that the Tm5 murine melanoma cell line constitutively and uniquely expresses a functional B1 receptor, while the B2 receptor is not present. We took advantage of this finding to address the particular contribution of the B1 receptor in tumor progression.

After Tm5 cells stimulation with the B1 receptor agonist DABK we observed increased intracellular calcium concentration and also increased levels of ERK phosphorylation, but no alteration in cell proliferation. Previous reports using systems in which both receptors are expressed, describe an induction of cell proliferation, which suggests that the B2 receptor may be required for pro-proliferative effects of DABK. Actually, we observed that *in vivo* tumors exposed to DABK presented several elements of decreased aggressiveness, including less proliferating Ki67 positive cells within the tumor mass. While the mechanisms underlying such *in vivo* inhibitory effects remains to be elucidated, the interface tumor/host seems to play a major role. One possibility is the immune system act as a key player by decreasing *in vivo* tumor proliferation, as we have observed changes in the cytokine profile within the tumor mass when comparing control and DABK-treated tumors. Furthermore, a significant decrease in melanoma cell migration was observed *in vitro*. The ability of the B1 receptor to disrupt cell migration has been previously described in non-neoplastic arterial smooth muscle cells [31], although this effect has not previously been reported in tumor cells. The mechanisms underlying the inhibition of cell migration are not yet clear. However, as previously reported [40] an increase in the expression of adhesion molecules, such as E-cadherin, can strongly decrease cell migration. Moreover, corroborating with previous data reporting a functional role for B1/B2 receptors cross talk [30], we show that introduction of B2 receptor in Tm5 cells completely abrogated the B1-mediated effect of decreasing cell migration, reinforcing a pivotal role for the B1/B2 receptors cross talk in cellular response and ultimately in tumor progression.

Our model also allowed us to specifically address whether the B1 receptor could play a role in tumor progression *in vivo*. In this sense, Tm5 cells were stimulated *in vitro* with DABK and 24 h later implanted into mice. This approach allowed us to evaluate the contribution of the B1 receptor without the interference of a cross talk with the B2 receptor. This aspect is especially important considering that the agonists of both receptors differ only by the presence or absence of a C-terminal arginine residue [10]. Remarkably, our data show that implantation of B1 receptor-stimulated cells resulted in a decreased the incidence of tumor formation. Besides that, tumors generated from B1 receptor-stimulated cells exhibited a significant decrease of inflammatory cells infiltration. This result is particularly interesting because in many solid tumors, the presence of a high number of inflammatory cells within the tumor area correlates with a poor prognosis [52]. Moreover, corroborating with a decreased incidence of tumor formation, we found that within the tumor mass, which includes tumor and host cells, mRNA levels of two pro-inflammatory cytokines, IL-6 and IFN-γ, were up-regulated in tumors from B1 receptor-stimulated cells compared to controls. Although inflammation has been shown to be a hallmark of cancer [53], and several pro-inflammatory cytokines play a role in tumor progression [54], IFN-γ and IL-6 have been described as key elements in host anti-tumor immune response [42]. It may be possible that activation of the B1 receptor, by inducing secretion of cytokines and chemokines, could attract and activate immune cells within the tumor area. In this sense, lymphocytes would be able to successfully elicit an anti-tumor immune response, which then could explain the higher levels of IFN-γ observed in the tumor mass of tumors from DABK-treated cells [42]. On the other hand, lymphocytes recruited to tumors from the control group showed to be associated with lower levels of IFN-γ. This allow us to speculate that tumor cells possibly were able to evade immune response by inducing anergy and/or senescence of T cells [55], or even inducing differentiation of T cells into T regulatory cells, which would then lead to a down regulation of immune response. In fact this hypothesis seems fairly coherent with the increased levels of TGF-β observed in control tumors when compared with tumors originated from DABK-treated cells, as it is known that tumor cells as well as regulatory T cells secrete TGF-β to inhibit immune response [56].

Tumors originated from DABK-stimulated cells also displayed patterns of decreased aggressiveness, such as fewer proliferating cells and a poorer vascular network. Another feature of aggressiveness is the ability of tumor cells to detach from the primary tumor and reach the circulation to colonize secondary organs and metastasize [2]. One of the required steps for a tumor cell to metastasize is to acquire a more motile and less adhesive phenotype. Therefore, we hypothesized that tumors originated from DABK-stimulated cells would also display reduced metastasis ability. In this sense, we observed that DABK-stimulated tumors had significantly higher levels of the adhesion molecule E-cadherin. Placing these findings in context, they suggest that activation of the B1 receptor by its agonist reprogrammed melanoma cells, which then generated less aggressive primary tumors, less prone to metastasize, and that ultimately improved animal survival.

Several studies have shown that ACE (as also known as kininase II) inhibition resulted in anti-tumor effects. [12]–[13]. Indeed, ACE is a key functional point connecting the renin-angiotensin system and the kallikrein-kinin system, being responsible for AngII formation as well as for kinin degradation [19]–[20]. Therefore, any inhibition/impairment in ACE functionality ultimately leads to a decrease in AngII levels and to an increase in kinin availability. In this context, it seems discordant that ACE blockade could play its anti-tumor role by increasing the activity of a pro-tumor system, i.e., the kallikrein-kinin system. Hence, it is tempting to speculate that the anti-tumor properties of ACE inhibitors might not be exclusively mediated by impairment of the renin-angiotensin system axis, but in fact may also involve the kallikrein-kinin system axis.

Taken together, our results show that activation of the GPCR kinin B1 receptor plays a host protective role during Tm5 melanoma progression in mice. The B1 receptor stimulation decreases melanoma cell migration and decreases tumor growth, proliferation, vascularization and immune cell infiltration, while increasing the expression of the pro-inflammatory and anti-tumor cytokines IFN-γ and IL-6. All these factors contributed to the complete absence of lymph node metastases and improved animal survival. The identification of a new GPCR with anti-tumor properties opens new avenues for the development and discovery of new potential pharmacological targets to treat tumor growth and dissemination, such the use of selective non-peptide B1 receptor agonists. These findings also have major implications for ACE inhibitors, which are widely used to treat hypertension, and their effects on a concomitant tumor disease, which require further investigation.

## Supporting Information

Figure S1 **Activation of the kinin B1 receptor inhibits cell migration of B16F10 melanoma cells** ***in vitro*** **.** (A) B16F10 melanoma cells express B1 receptor, but do not express kinin B2 receptor. B1 receptor activation decreases collective (B–C) and single cell migration (B and D) in B16F10 melanoma cell line. DABK: desArg^9^-bradykinin; n = 3 independent experiments performed in triplicate; Data are expressed as the mean ± SEM; **** p<0.0001. The scale bars represent 200 μm.
(TIF)

Table S1 **Primers sequences, temperature of melting and fragment size obtained in semi-quantitative PCR (sqPCR) and/or quantitative PCR (qPCR).** Bp: base pairs; Tm: temperature of melting; CPM: carboxypeptidase M; TGF-β: transforming growth factor beta; INF-γ: interferon-gamma.
(DOCX)
