# Supplementary figures and images for: Activation of the Kinin B1 Receptor Attenuates Melanoma Tumor Growth and Metastasis
Source: PLoS One. 2013 May 17;8(5):e64453. doi: 10.1371/journal.pone.0064453 (PMC3656876; doi:10.1371/journal.pone.0064453)

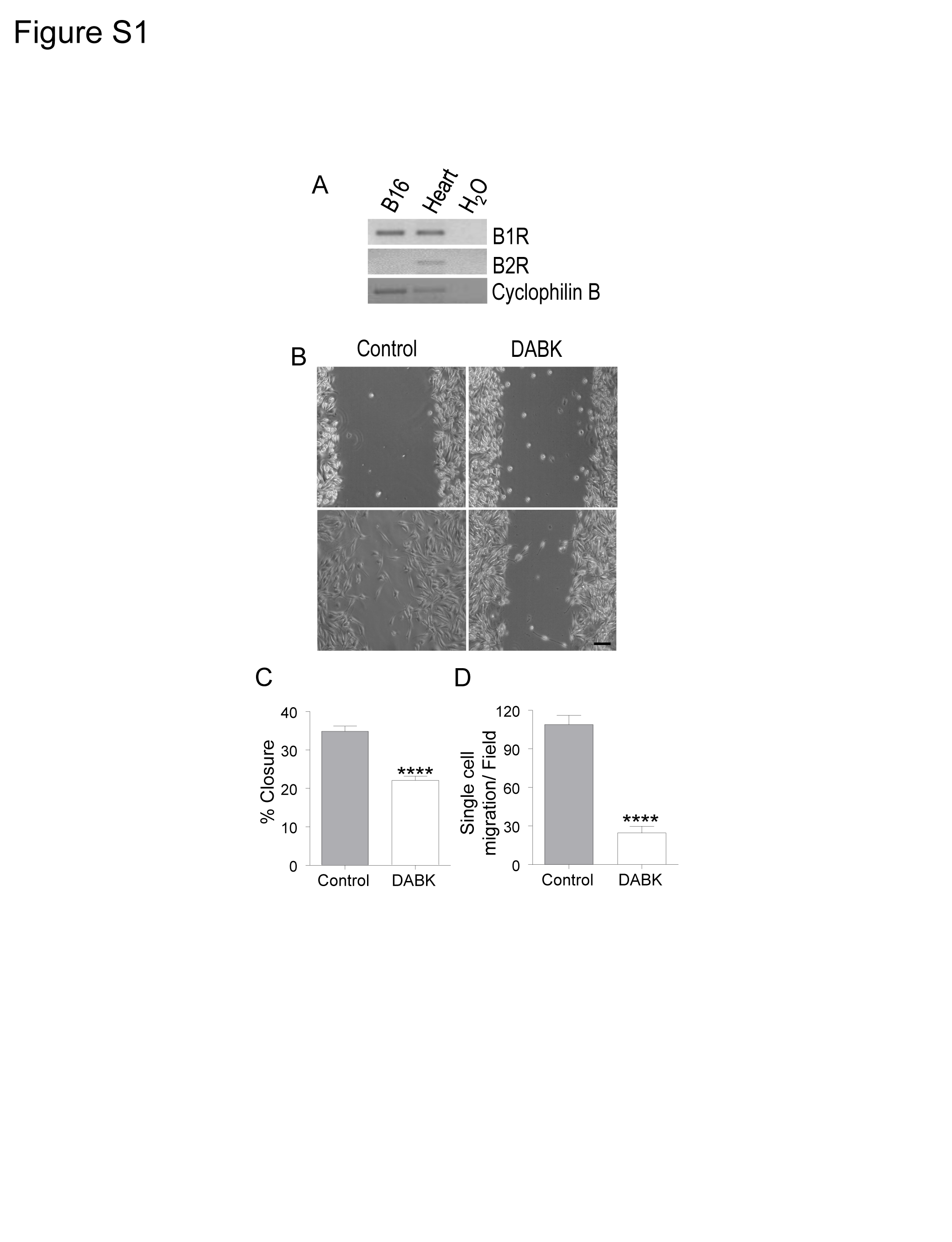

Supplement: Figure S1 — Activation of the kinin B1 receptor inhibits cell migration of B16F10 melanoma cells in vitro . (A) B16F10 melanoma cells express B1 receptor, but do not express kinin B2 receptor. B1 receptor activation decreases collective (B–C) and single cell migration (B and D) in B16F10 melanoma cell line. DABK: desArg9-bradykinin; n = 3 independent experiments performed in triplicate; Data are expressed as the mean ± SEM; **** p<0.0001. The scale bars represent 200 μm. (TIF) [file pone.0064453.s001.tif]
