# Supplementary material for: Activation of the Kinin B1 Receptor Attenuates Melanoma Tumor Growth and Metastasis
Source: PLoS One. 2013 May 17;8(5):e64453. doi: 10.1371/journal.pone.0064453 (PMC3656876; doi:10.1371/journal.pone.0064453)
Supplement: Table S1 — Primers sequences, temperature of melting and fragment size obtained in semi-quantitative PCR (sqPCR) and/or quantitative PCR (qPCR). Bp: base pairs; Tm: temperature of melting; CPM: carboxypeptidase M; TGF-β: transforming growth factor beta; INF-γ: interferon-gamma. (DOCX) [file pone.0064453.s002.docx]

**Table S1:** Primers sequences, temperature of melting and fragment size obtained in semi-quantitative PCR (sqPCR) and/or quantitative PCR (qPCR). Bp: base pairs; Tm: temperature of melting; CPM: carboxypeptidase M; TGF-β: transforming growth factor beta; INF-γ: interferon-gamma.

| **Target** | **PCR** | **Tm**  **(°C)** | **Forward (5'-3')** | **Reverse (5'-3')** |
| --- | --- | --- | --- | --- |
| **Cyclophilin B** | sqPCR/qPCR | 55 | AAA GAC TTC ATG ATC CAG GG | TGA CAT CCT TCA GTG GCT TG |
| **B_1_ receptor** | sqPCR | 55 | CAC GAA GCT TGG CAC TTT GT | GTC TGT GAG CTC CTT CCA GAA |
| **B_2_ receptor** | sqPCR | 56 | GCA CTG TGG CCG AGA TCT A | GCT GTA TTC CCT CAT GGT CCT |
| **CPM** | sqPCR | 55 | AAA CAT TTG TCC TCT CTG CGA | TGT AGG CCA GGT GTT GGA AA |
| **E-cadherin** | qPCR | 56 | GCC AAT CCT GAT GAA ATT GGA A | CAG AAC CAC TGC CCT CGT AAT C |
| **TGFβ** | qPCR | 55 | GCA ACA TGT GGA ACT CTA CCA G | CAG CCA CTC AGG CGT ATC A |
| **IFNγ**  **B1 receptor** | qPCR  qPCR | 55  62 | CAG CAA CAG AAG GCG AAA AAG G  TCT GGG ATA GAC CAC AGC T | AAT CTC TTC CCC ACC CCG AAT CA  TGC TGG CTT TGG TTA GAA GG |
| **GAPDH** | sqPCR/qPCR | 55 | GAA GGT GAA GGT CGG AGT C | GAA GAT GGT GAT GGG ATT TC |
